# Effects of diabetes self-management education program on lowering blood glucose level, stress, and quality of life among females with type 2 diabetes mellitus in Thailand

**DOI:** 10.1017/S1463423621000505

**Published:** 2021-09-15

**Authors:** Monthalee Nooseisai, Pramon Viwattanakulvanid, Ramesh Kumar, Napaphan Viriyautsahakul, Gul Muhammad Baloch, Ratana Somrongthong

**Affiliations:** 1 College of Public Health Sciences, Chulalongkorn University, Bangkok, Thailand; 2 Department of Health, Bangkok Metropolitan Administration, Bangkok, Thailand; 3 Department of Public Health, Health Services Academy, Islamabad, Pakistan; 4 Department of Public Health, School of Medicine, Taylor’s University, Selangor, Malaysia

**Keywords:** diabetes management, education program, HbA1c and health education, quality of life, stress

## Abstract

**Aim:**

The aim of this study is to assess the effect of diabetes self-management education (DSME) on lowering blood glucose level, stress, and quality of life (QoL) among female patients with type 2 diabetes mellitus (T2DM) in Thailand.

**Background:**

The burden of noncommunicable diseases has increased globally, and it has negatively affected the QoL of diabetic patients.

**Methods:**

A quasi-experimental study was conducted by including 77 T2DM patients selected from 2 public health centers in Thailand. The respondents were randomly selected 38 in control group and 39 in intervention group. Pretested, piloted, and validated tool were used during this study. Knowledge on blood glucose level, stress, and QoL was measured at baseline and then compared to end line after 3 months of the intervention. The effects of intervention were estimated by regression coefficient of intervention on blood glucose level and QoL. The study was ethically approved by the Chulalongkorn University, Thailand.

**Findings:**

Baseline characteristics of both the groups were similar before the start of the intervention and there were no significant differences observed in age, education, blood sugar monitoring behavior, medical checkup, knowledge, self-care, stress, and hemoglobin HbA1c (>0.05). However, blood HbA1c, stress level, and QoL among the T2DM patients had significant changes (<0.05) after the intervention. The control group was remained same and there was no statistically significant difference reported (>0.05).

**Conclusions:**

The study concluded that the designed intervention of DSME has proved effective in lowering the blood sugar level, HbA1c level, stress level, and improved QoL among T2DM patients during this limited period of time. Hence, policy-makers can replicate this intervention for diabetic patients in a similar context.

## Introduction

Diabetes mellitus (DM) is considered a major public health problem that badly affects the quality of life (QoL) of patients (Inga-Britt & Kerstin, 2019). Thailand is one of the countries reporting high number of aging population and increasing the burden of noncommunicable disease (Somanawat *et al.*, 2020). High prevalence of DM is badly affecting the health of patients and resulting in multiple complications with poor QoL (Abedini *et al.*, 2020). QoL is one of the most important factors that contributes to treatment outcomes of self-assessing the effects of the management of diabetes. QoL is highly influenced by personal expectations, attitudes, practices, and knowledge of the patient for that particular disease (Borg *et al.*, 2019). In this condition, endocrine system releases excess glucocorticoids hormone that would impair glucose production in the liver and reduce the sensitivity of the cells for insulin that causes hyperglycemia (Di Dalmazi *et al.*, 2012).

Prevalence of stress is very high among diabetic patients, especially women patients having quite higher numbers than male (Aekplakorn *et al.*, 2018). Diabetes-related distress is a syndrome with multiple symptoms like anxiety, conflict, frustration, and confusion among the diabetic patients (Tunsuchart *et al.*, 2020). Stress among the women can be reduced through their active involvement in routine work like crocheting at home (Guo *et al.*, 2019; Abedini *et al.*, 2020). Bangkok is an area with high prevalence of diabetes at 7.3 % of population aged 15 years or more (Aekplakorn, 2016), and only 47.7% of type 2 diabetes mellitus (T2DM) patients had HbA1c less than 7 (Rungsin & Thasanawiwat 2015). Stress is affecting most of the diabetic patients due to their chronic nature of this disease (Ahangari *et al.*, 2016). Stress might affect the behavior of chronic DM patients with emotional mood disturbances. There is a negative correlation found between stress and treatment adherence level among type 2 DM patients because of lack of awareness and carelessness (Vasanth *et al.*, 2017). Hence, this uncontrolled glycemic control may develop long-term complications, stress, and impacts on the QoL among DM patients (Miftari & Melonashi, 2015; Perrin *et al.*,^2017^).

Diabetes self-management education (DSME) is an important program for patients in managing their sugar level on regular basis and has proved as an effective approach (Chrvala *et al.*, 2016). This coping strategy is essential for effective self-care in diabetes (Kent *et al.*, 2010). Many studies have shown positive results of this intervention approach (Kelly *et al.*, 2012; Zamani-Alavijeh *et al.*, 2018). Mindfulness-Based Stress Reduction (MBSR) is a health-giving method that combines relaxation and physical activity. This could be more beneficial for the patients suffering from the chronic illness (Armani Kian, 2018). A study by Pollanen *et al.* confirmed that the craft activities were commonly seen in providing recreation and satisfaction for better feelings in depression (Pollanen *et al.*, 2015). Textile hobby crafts like knitting and crocheting are another popular leisure activities for reducing the stress (Kouhia, 2016; Kaimal *et al.*, 2017; Maritz, 2017; Daisy *et al.*, 2019). It has been shown that knitting site has proved an effective stress reliever among majority of patients, and it has also been reported that knitting helped them to cope up with emotional mood (Riley *et al.*, 2016; Burns & Van Der Meer 2020).

As diabetes situation, continues to worsen over time, especially among women, most patients face difficulty in controlling their blood glucose level. DSME is an important strategy to help DM patients in managing their health conditions. This strategy has never been tested in Thai patients to control their blood sugar. However, alternative intervention choices are needed for each patient group and lifestyle. With a potential benefit as recreational activity for stress coping method, crocheting practice program was incorporated in the DSME. This study aims to evaluate the effect of DSME on blood glucose level among adult female patients with T2DM in public health centers in Bangkok.

## Methods

This was quasi-experimental study design conducted from January to June 2020. T2DM patients were selected randomly from the list of register available with two public health centers of Bangkok. Thonburi was selected as control and Nongkham was selected as intervention arm. These centers were selected from the two regions providing health services to the population of similar sociodemographic characteristics. Sample size of 77 respondents was calculated by taking the intervention effect at 20% (Faul *et al.*, 2009; Kanghae, 2015). Respondents were randomly selected from both centers and assigned 38 in control and 39 in intervention arm. T2DM patients with the age of 50–65 years, female by gender, non-insulin-dependent, HbA1c >7% during last 3 months were included in the study. However, those with hypoglycemia, associated complications, eyesight problems, and disability were excluded from the study. Pretested, piloted, and validated tool were used during this study. Cronbach alpha coefficient was measured as 0.8 and two experts also assessed the construct validity, index of item-objective congruence (IOC) as .80. World Health Organization Quality of Life-BREF (WHOQOL-BREF) tool was adopted to assess the QoL among Thai population (World Health Organization, 1996; Mahannirankul *et al.*, 1998). Stress Test-20 (SPST – 20) was used for the stress measurement and the questionnaire was designed to measure two components of mindfulness: awareness and acceptance (Mahanirankul *et al.*, 1997, Cardaciotto *et al.*, 2008). Blood glucose level and HbA1_c_ were measured for average level of plasma glucose over the previous 2–3 months and analyzed by turbid metric inhibition immuno assay. Height and weight were used to check the BMI of the participants. Sociodemographic information and medical history information were collected at the time of interview. Mean scores of QoL were calculated and grouped in three levels: low, moderate, and high. The questionnaire comprised of two types of questions: perceived objective and self-report subjective questions for four facets: physical health, psychological health, social relationships, and environment and two items relating to the overall QoL and general health. The scores of each question ranged from 1 to 5; higher the score means the higher is the QoL. Data were analyzed by statistical package for social science (SPSS version 22). Descriptive statistics like percentage and frequency, and mean and standard deviation (*SD*) were used, and inferential statistics like the Shapiro–Wilk or Kolmogorov–Smirnov test, Mann–Whitney U test, Wilcoxon-signed rank test, and parametric statistics such as independent t-test, correlation, multiple linear regression, and independent t-test were used to compare the groups. Chi-square test was used to test the association among categorical variables. Hierarchical regression was used to see the effect of intervention. To adjust for the confounding factors (covariates), the difference and difference (DID) was (ê) used. The study was reviewed and approved by the Research Ethics Review Committee for Research Involving Human Research Participants, Health Sciences Group, Chulalongkorn University (Ref. COA No. 235/2559). Participants were also provided with information on the objectives and study procedures, and written consents were obtained prior to participation in the study.

## Intervention

The DSME was adopted from successive literature and reliable interventions conducted in different parts of the world (Rungsin & Thasanawiwat, 2015; Riley etal., 2016; Aekplakorn *et al.*, 2018; Daisy & Saoirse, 2019; Burns & Van Der Meer, 2020). All contents were prepared and printed for T2DM patients and distributed to participants. The curriculum was compiled with basic knowledge about diabetes, the importance of self-management and self-care. A total of 8 h of direct contact sessions were performed. Each 2-h monthly session continued for 4 months of the intervention. These sessions included presentations, discussions, demonstrations, and real experiences with examples. The trained team, with background knowledge in health, delivered intervention. Participants were asked for crocheting practice, and assignment was given to conduct for 3 days a week. This aspect is a very important stress relief intervention, required by women to do at their home. Yarn and needle were provided at the end of each class for participants for real exercise in the class and to continue this activity at their home.

## Results

The average age of participants was 58.86 (±4.73) years, suffering from T2DM 7.68 (±4.16) years; average HbA1c level was 8.26 (±0.74) years. Both groups were selected from two different areas with similar basic characteristics and assessed at baseline before the intervention. There was no significant difference between groups for age, education, blood sugar monitoring behavior, medical checkup, DM knowledge, self-care, stress, and HbA1c. Hence, both groups had same characteristics except occupation, exercise, and BMI at baseline (Table 1).

**Table 1.** Participants’ characteristics between control and intervention groups at baseline

Variables		Total (*N* = 77)	Control (*N* = 38)	Intervention (*N* = 39)	*p*-Value
		Mean ± SD or *n* (%)	Mean ± SD or *n* (%)	Mean ± SD or *n* (%)	
Age	(Mean ± SD)	58.86 ± 4.73	58.82 ± 4.32	58.9 ± 5.15	0.941^ϵ^
	60 yr. >	39 (50.6)	18 (47.4)	21 (53.8)	0.570^#^
	< 60 yr.	38 (49.4)	20 (52.6)	18 (46.2)	
Education	Primary	61 (79.2)	33 (86.8)	28 (71.8)	0.104^#^
	<Primary	16 (20.8)	5 (13.2)	11 (28.2)	
Occupation	House wife	42 (54.5)	14 (36.8)	28 (71.8)	0.002^# *^
	Working	35 (45.5)	24 (63.2)	11 (28.2)	
Exercise	No exercise	30 (39.0)	21 (55.3)	9 (23.1)	0.004^#*^
	Sometime exercise	47 (61.0)	17 (44.7)	30 (76.9)	
Blood sugar monitoring	Occasionally	31 (40.3)	18 (47.4)	13 (33.3)	0.209^#^
	Never monitored	46 (59.7)	20 (52.6)	26 (66.7)	
Medical checkup	Always	71 (92.2)	37 (97.4)	34 (87.2)	0.095^#^
	Sometimes	6 (7.8)	1 (2.6)	5 (12.8)	
Health status	BMI (Mean ± SD)	26.62 ± 4.01	27.66 ± 4.39	25.61 ± 3.37	0.024^ϵ^*
	DM (Mean ± SD)	7.68 ± 4.16	8.53 ± 4.76	6.85 ± 3.35	0.078^ϵ^
	HbA1c (Mean ± SD)	8.26 ± 0.74	8.32 ± 0.758	8.21 ± 0.729	0.510^ϵ^
Knowledge		12.16 ± 1.51	12.03 ± 1.37	12.28 ± 1.64	.460^ϵ^
Self-care		71.69 ± 7.15	72.42 ± 4.69	70.97 ± 8.92	.375^ϵ^
Stress		35.04 ± 9.47	36.03 ± 10.20	34.08 ± 8.71	0.530 u
Low		8 (10.4)	3 (7.9)	5 (12.8)	0.882 a
Moderate		50 (64.9)	25 (65.8)	25 (64.1)	
High		19 (24.7)	10 (26.3)	9 (23.1)	
Quality of life		91.10 ± 11.43	89.55 ± 7.09	92.62 ± 14.39	0.240 u

*Statistically significant (*p* < 0.05), ^#^Chi-square test, ^ϵ^Independent t-test, a: Fisher’s exact test, u Mann–Whitney *U* test.

Results show that the control and intervention groups are significantly different in three important aspects: control of blood glucose level, mindfulness-based stress, and improved QoL. Mindfulness-based stress means relaxation activity among diabetic patients do crochet at home that was found effective in reducing the stress level among them (*P* <0.001). This intervention positively affected the control of blood glucose level among the participants as compared to control intervention (*P* <0.001). Hence, the blood HbA1c level in intervention group significantly decreased in the intervention arm and increased in the control arm (*P* = 0.008). QoL among diabetic patients was measured through mean score that increased in the intervention group but decreased in the control group. The change of QoL was significantly different between control and intervention group (*P* = 0.042). However, control group was observed during this period and remained same for the blood glucose level, mindfulness-based stress, and QoL among the participants. Both groups were geographically located in two different areas and there was no chance of intervention contamination during this research. (Table 2).

**Table 2.** Comparison of knowledge, self-care, stress, HbA1_C_, and Qol in both groups

Variables		Total (*N* = 77)	Control group (*N* = 38)	Intervention group (*N* = 39)	*p*-Value
		Mean ± SD or *n* (%)	Mean ± SD or *n* (%)	Mean ± SD or *n* (%)	
Stress	Low stress group	17(22.1)	3 (7.9)	14 (35.9)	0.007^#*^
	Moderate stress group	45(58.4)	28 (73.7)	17 (43.6)	
	High stress group	15(19.5)	7 (18.4)	8 (20.5)	
Mindfulness	Component 1: Mindfulness	24.29 ± 8.95	18.24 ± 4.81	30.21 ± 8.08	<0.001^ϵ^*
	Mindfulness	.07 ± 10.63	−4.74 ± 7.31	4.74 ± 11.33	<0.001^ϵ^*
	Component 2: Acceptance	37.45 ± 9.27	43.39 ± 4.78	31.67 ± 8.93	<0.001^ϵ^*
	Acceptance	−1.03 ± 9.92	2.92 ± 6.46	−4.87 ± 11.21	<0.001^ϵ^*
Blood glucose level	HbA1_c_	8.11 ± .88	8.38 ± 1.036	7.84 ± .613	0.008^ϵ^*
	▵ HbA1_c_	−.13 ± .62	0.056 ± .77	−.36 ± .50	0.006^ϵ^*
Quality of life	Quality of life	89.79 ± 10.49	85.87 ± 6.75	93.62 ± 12.05	<0.001 u*
	Good	22 (28.6)	5 (13.2)	17 (43.6)	0.003 ^#^*
	Moderate	55 (71.4)	33 (86.8)	22 (56.4)	

*Statistically significant (*p* < 0.05), ^ϵ^independent t-test, ^#^Chi-square test, u: Mann–Whitney *U* test, ê difference in difference.

To estimate the effect of intervention on the change of HbA1c, hierarchical regression analysis was used. In model 1, the change of HbA1c is taken as dependent variable, and intervention was independent variable. Simple linear regression showed significant negative association between intervention and outcome (*β* = −0.304, *P* = 0.007) *R* ^2^ was 0.092. Based on the bivariate analysis, we selected covariates to put in model 2 and 3. In model 2, we adjusted stress, the change of mindfulness, and acceptance as covariates. Other factor in control, and intervention groups is the change of mindfulness was statistically significant associated with the change of HbA1c (*β* = −0.394, *P* = 0.002 and *β* = 0.629, *P* = 0.018; respectively) *R* ^2^ was .167. In model 3, we added more covariates: occupation, exercise, BMI, and R^2^ increased to .188. Same as in model 2, only intervention program and the change of mindfulness had statistically significant association with the change of HbA1_c_ (*β* = −0.494, *P* = 0.002 and *β* = 0.695, *P* = 0.020) (Table 3).

**Table 3.** The effect of the intervention program on with the change of HbA1_c_ with hierarchical regression analysis

Model	Factors	Unstandardized coefficients		Standardized Coefficients	*p*-Value#	95.0% CI for b	
		*b*	SE	*β*		LB	UB
1	(Constant)	.056	.096		0.562	−.135	.247
	Intervention	−.372	.135	−.304	0.007*	−.640	−.104
2	(Constant)	−.009	.239		.969	−.486	.467
	Intervention	−.481	.148	−.394	.002*	−.776	−.187
	▵ Mindfulness	.036	.015	.629	.018*	.007	.066
	▵ Acceptance	.028	.015	.447	.077	−.003	.059
	Stress	.074	.107	.078	.488	−.139	.288
3	(Constant)	.371	.625		.554	−.875	1.618
	Intervention	−.605	.190	−.494	.002*	−.983	−.226
	▵ Mindfulness	.040	.017	.695	.020*	.007	.074
	▵ Acceptance	.029	.017	.475	.083	−.004	.063
	Stress	.069	.112	.073	.541	−.155	.294
	Occupation	.103	.168	.081	.544	−.233	.439
	Exercise	−.104	.188	−.066	.582	−.480	.271
	BMI	.081	.153	.065	.596	−.223	.386

^#^Multiple linear regression, *statistically significant *p* < 0.05, *R* ^2^ (Model 1) = 0.092, *R* ^2^ (Model 2) = 0.167, *R* ^2^ (Model 3) = 0.188, ê difference in difference.

## Discussion

Patients in intervention group had significant reduction of HbA1c after receiving the education program as compared with control group, indicating that the intervention had positive effects for improving the health status of patients with type 2 diabetes. An intervention study from Thailand is also concurrent with findings of this study and proved that diabetes and blood sugar level can be controlled through self-management (Hurst *et al.*, 2020). A study on self-monitoring of blood glucose in adults with type 2 diabetes reported the same results with positive effect of intervention (Ward *et al.*, 2015). Blood HbA1c control has been shown to be one of the most important clinical outcomes among diabetic patients, and the control of blood glucose has been shown to be positive for the prevention of diabetic complications in Thai population (Sieng *et al.*, 2015). Another survey by Polonsky *et al.*, (2011) also supported findings of this study. Yet another study shows that factors like blood sugar self-monitoring method, negative emotion, lack of motivation, busy schedule, and cost of monitoring could affect the levels of HbA1c (Lawal *et al.*, 2017). Local research has also supportive evidence for the finding of this study (Somanawat *et al.*, 2020). For health status, compared with control group, intervention group had lower BMI, with lower HbA1c levels. These characteristics are relevant to previous research, which showed correlation between HbA1c, BMI, and DM complication (Gray *et al.*, 2015; Babikr *et al.*, 2016). Most of the participants in intervention group were housewives, and sedentary, but had more exercise; while most of the control group participants had other occupations, and although more active in physical activity, but had less exercise, which may be due to their occupations. Work was reported as one of the barriers to exercise (Korkiakangas *et al.*, 2011). A systematic review and meta-analysis showed positive benefit of intervention on outcomes such as blood sugar control and psychosocial outcomes (Davies *et al.*, 2018). Another study also supports findings of this study that an intervention can integrate with stress and blood sugar level among respondents (Daisy & Saoirse, 2019). Yet other study has showed that mindfulness intervention reduced blood sugar level in T2DM patients (Vala *et al.*, 2016; Armani Kian, 2018).

Intervention group had a decrease in stress and improvement in QoL after receiving the education program compared with control group, indicating that the intervention had positive effects in reducing stress and improving QoL among patients with type 2 diabetes. These findings are also consistent with study conducted in Thailand (Tunsuchart *et al.*, 2020). Another study supported findings of this study with similar participants’ stress profile (Sulukananuruk *et al.*, 2016). Crochet intervention showed positive results in improving the QoL among the participants and also being effective in stress reduction (Riley *et al.*, 2016; Burns & Van Der Meer, 2020). After intervention, this study found that a number of participants in low stress group increased from 12.8% to 35.9% in intervention group, while it was stable in control group at 7.9%. This is the possible effect of textile crafting as leisure-based coping strategy used in the intervention. This helps to manage stress through the production of an artifact and by giving peaceful time and intellectual work in participants. Study by Pollanen *et al.*, (2015) also supports finding of this study that this continuous activities among the patients could positively reduce their stress level.

This study found the positive effect of self-management education and crocheting program on QoL. This was an effective and encouraging exercise for the women to relieve their stress level at their home. After intervention, the average score of QoL increased in the intervention group as compared to control arm. Findings of this study are consistent with results of other studies and reported benefit of DSME on QoL (Daisy & Saoirse, 2019; Abedini *et al.*, 2020). A study conducted from the same country (Thailand) also supports findings of this study (Tunsuchart *et al.*, 2020). Moreover, few more studies have also proved that stress reduction has positive impacts on QoL among the participants (Guo *et al.*, 2019; Abedini *et al.*, 2020). Study design and the intervention effectiveness on diabetes self-management could be considered as the major strengths of this study. The limitations of this study were the time constraints and a combination of two interventions that cannot disentangle their separate effects. However, the findings of this study cannot be generalized for the whole country.

## Conclusion

Our results indicated that DSME has positive effects on lowering blood glucose level, reducing stress, and improving QoL among adult female patients with type 2 diabetes during this limited period of time. Hence, this intervention can be usefully replicated for the diabetic patients in the similar context with high prevalence of diabetes.
